# ESPO/ BIOLOGICAL SCIENCES SECTION SYMPOSIUM: EMERGING INSIGHTS IN INTERACTIONS AND NETWORKS IN THE BIOLOGY OF AGING DEVELOPING C. ELEGANS TO STUDY AGE-RELATED EXTRACELLULAR VESICLE SIGNALS

**DOI:** 10.1093/geroni/igz038.1583

**Published:** 2019-11-08

**Authors:** Joshua Russell, Matt Kaeberlein

**Affiliations:** University of Washington, Seattle, Washington, United States

## Abstract

All cells release vesicles into their extracellular environment. These extracellular vesicles (EVs) contain multiple classes of molecules, including nucleic acids, proteins, and lipids. EV-signaling has been shown to be impacted by many age-related physiological processes such as inflammation, mitochondrial stress, and autophagy as well as directly mediate critical functions in cellular senescence and aging. The isolation and analysis of EV cargos from mammalian cell culture and liquid biopsy samples has become a powerful approach for uncovering the messages that are packaged into these organelles. Caenorhabditis elegans is a premier model for dissecting the genetics of aging however, EV analysis has not been tenable in invertebrate model systems due to lack of methods for obtaining sufficient amounts of pure EVs. We developed a method for isolating pure EVs from C. elegans with yields sufficient for mass spectrometry and RNAseq. Here we present the analysis of the genetic and protein cargos of EVs collected from wild type and long-lived mutants collected at different time points across their lifespans. As the first investigation of age-related EV signals in an invertebrate model system we believe these results will provide insights into cell non-autonomous mechanisms of aging.

